# O-Glycosylation Regulates Ubiquitination and Degradation of the Anti-Inflammatory Protein A20 to Accelerate Atherosclerosis in Diabetic ApoE-Null Mice

**DOI:** 10.1371/journal.pone.0014240

**Published:** 2010-12-06

**Authors:** Gautam V. Shrikhande, Salvatore T. Scali, Cleide G. da Silva, Scott M. Damrauer, Eva Csizmadia, Prabhakar Putheti, Michaela Matthey, Roy Arjoon, Rakesh Patel, Jeffrey J. Siracuse, Elizabeth R. Maccariello, Nicholas D. Andersen, Thomas Monahan, Clayton Peterson, Sanah Essayagh, Peter Studer, Renata Padilha Guedes, Olivier Kocher, Anny Usheva, Aristidis Veves, Elzbieta Kaczmarek, Christiane Ferran

**Affiliations:** 1 The Division of Vascular Surgery, Department of Surgery and the Center for Vascular Biology Research, Department of Medicine, Beth Israel Deaconess Medical Center, Harvard Medical School, Boston, Massachusetts, United States of America; 2 The Transplant Institute, Department of Medicine, Beth Israel Deaconess Medical Center, Harvard Medical School, Boston, Massachusetts, United States of America; 3 The Department of Pathology, Department of Medicine, Beth Israel Deaconess Medical Center, Harvard Medical School, Boston, Massachusetts, United States of America; 4 The Division of Endocrinology, Department of Medicine, Beth Israel Deaconess Medical Center, Harvard Medical School, Boston, Massachusetts, United States of America; 5 Joslin-Beth Israel Deaconess Foot Center and Microcirculation Laboratory, Department of Medicine, Beth Israel Deaconess Medical Center, Harvard Medical School, Boston, Massachusetts, United States of America; 6 The Transplant Institute, Department of Medicine, Beth Israel Deaconess Medical Center, Harvard Medical School, Boston, Massachusetts, United States of America; 7 Division of Nephrology, Department of Medicine, Beth Israel Deaconess Medical Center, Harvard Medical School, Boston, Massachusetts, United States of America; University of Padova, Italy

## Abstract

**Background:**

Accelerated atherosclerosis is the leading cause of morbidity and mortality in diabetic patients. Hyperglycemia is a recognized independent risk factor for heightened atherogenesis in diabetes mellitus (DM). However, our understanding of the mechanisms underlying glucose damage to the vasculature remains incomplete.

**Methodology/Principal Findings:**

High glucose and hyperglycemia reduced upregulation of the NF-κB inhibitory and atheroprotective protein A20 in human coronary endothelial (EC) and smooth muscle cell (SMC) cultures challenged with Tumor Necrosis Factor alpha (TNF), aortae of diabetic mice following Lipopolysaccharide (LPS) injection used as an inflammatory insult and in failed vein-grafts of diabetic patients. Decreased vascular expression of A20 did not relate to defective transcription, as A20 mRNA levels were similar or even higher in EC/SMC cultured in high glucose, in vessels of diabetic C57BL/6 and FBV/N mice, and in failed vein grafts of diabetic patients, when compared to controls. Rather, decreased A20 expression correlated with post-translational O-Glucosamine-N-Acetylation (O-GlcNAcylation) and ubiquitination of A20, targeting it for proteasomal degradation. Restoring A20 levels by inhibiting O-GlcNAcylation, blocking proteasome activity, or overexpressing A20, blocked upregulation of the receptor for advanced glycation end-products (RAGE) and phosphorylation of PKCβII, two prime atherogenic signals triggered by high glucose in EC/SMC. A20 gene transfer to the aortic arch of diabetic ApoE null mice that develop accelerated atherosclerosis, attenuated vascular expression of RAGE and phospho-PKCβII, significantly reducing atherosclerosis.

**Conclusions:**

High glucose/hyperglycemia regulate vascular A20 expression via O-GlcNAcylation-dependent ubiquitination and proteasomal degradation. This could be key to the pathogenesis of accelerated atherosclerosis in diabetes.

## Introduction

Diabetic macrovasculopathy (DV), an accelerated form of atherosclerosis, is the leading cause of morbidity and mortality in diabetes mellitus (DM). Diabetic patients suffer a 2 to 4-fold increase in the incidence of coronary artery disease and stroke and a >10-fold increase in the incidence of peripheral vascular disease [1]. This begs for a better understanding of the molecular basis for DV.

Multiple risk factors including insulin resistance, dyslipidemia, and hyperglycemia account for accelerated atherosclerosis in patients suffering from type II diabetes mellitus [2]. On the cellular level, endothelial (EC) and smooth muscle (SMC) cells accumulate intracellular glucose during hyperglycemic episodes [3], [4]. This leads to the generation of reactive oxygen species (ROS) by the mitochondrial electron transport chain [5], setting in motion a number of pro-atherogenic signals that culminate in the phosphorylation of PKCβII [6], generation of advanced glycation end-products (AGE) [7], and amplification of inflammatory responses through activation of NF-κB [5]. All of these processes contribute to vascular complications of diabetes [8]. Additionally, high glucose enhances glucose flux through the hexosamine biosynthetic pathway (HBP), increasing the conversion of glucose to UDP-*N* Acetylglucosamine (UDP-GlcNAc), the substrate required for protein O-GlcNAcylation [9]. O-GlcNAcylation acts as a glucose sensor in that it is a dynamic, reversible post-translational modification (PTM) that responds to extra-cellular stimuli [10], [11]. In the vasculature, O-GlcNAcylation tips the balance towards heightened atherogenesis by decreasing the function of atheroprotective proteins, such as endothelial nitric oxide synthase (eNOS), while increasing the transcription of pro-atherogenic genes, such as *thrombospondin-1* [12], [13], [14], [15].

A20 maps to an atherosclerosis susceptibility locus in mice, with a single point mutation resulting in diminished A20 function in atherosclerosis-prone C57BL/6 mice as compared to atherosclerosis-resistant FBV/N [16], [17]. Our group demonstrated that A20 plays a crucial role in preventing and reverting neointimal hyperplasia through its effects in both EC and SMC [18]. A20 protects EC from apoptosis and blocks inflammation by inhibiting NF-κB activation in response to a broad spectrum of pro-atherogenic activators [19], [20], [21]. On the molecular level, The NF-κB inhibitory function of A20 is supported by its ubiquitin-editing functions [22]. A20 exerts dual deubiquitinase and ubiquitin ligase enzymatic activities that target adaptor and signaling molecules such as receptor interacting protein (RIP) and TNF-R associated protein (TRAF-6) either promoting their proteasomal degradation or regulating their interactions with other signaling molecules. In fact, A20 is part of an ubiquitin-editing protein complex, which includes Ring domain protein (RNF11) and the regulatory molecule TAX1BP1, which is implicated in the disruption of ubiquitin enzyme complexes through ubiquitination and degradation of the E2 ubiquitin conjugating enzymes Ubc13 and UbcH5c [23], [24]. Importantly, A20 maintains its anti-inflammatory/NF-κB inhibitory function in SMC, blocking upregulation of the pro-atherogenic proteins inter cellular adhesion molecule (ICAM-1) and monocyte chemoattractant protein-1 (MCP-1) and inhibiting SMC proliferation [18]. Additionally, A20 sensitizes intimal SMC to apoptosis through a NO-dependent mechanism, promoting regression of established lesions of intimal hyperplasia [18]. In this work, we questioned whether glucose alters the expression/function of A20 in EC and SMC, and whether this impacts the incidence/progression of DV in a mouse model [19], [25]. Our results indicate that A20 undergoes specific glucose-triggered post-translational modifications (PTM) including O-GlcNAcylation and ubiquitination, which leads to its loss by proteasomal degradation, hence depriving the organism of a key element of its atheroprotective armamentarium and accelerating development of atherosclerotic lesions.

## Results

### High glucose decreases A20 protein levels in response to inflammatory stimuli in SMC and EC without affecting mRNA levels

**Figure 1:**
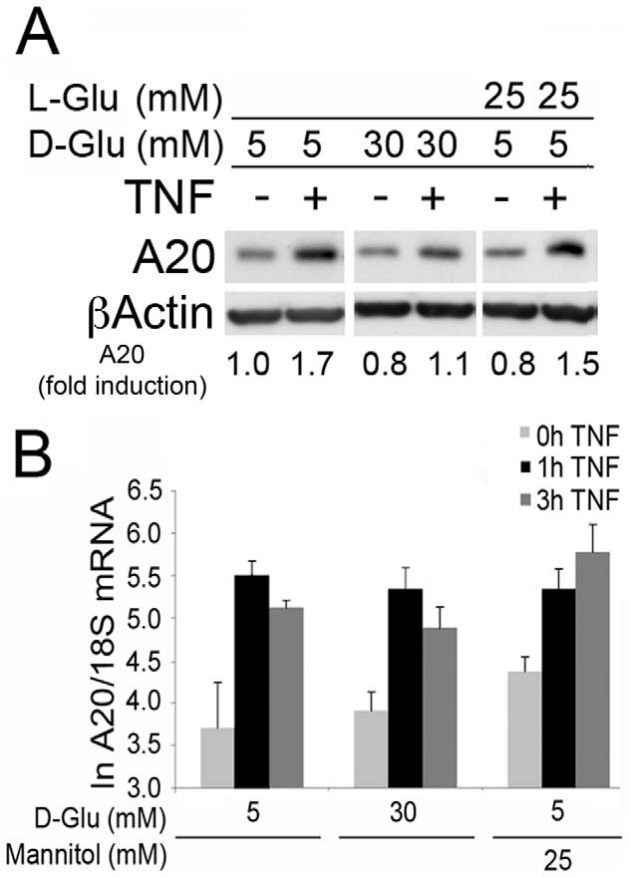
Increasing glucose (D-Glu) concentrations decreases TNF-mediated A20 protein up-regulation without affecting its transcriptional activation in SMC. (A) Analysis of A20 expression by WB. SMC were cultured in 5, or 30 mM D-Glu, or the osmotic control (25 mM L-Glu +5 mM D-Glu), and treated with TNF for 6 h. βactin was checked as a loading control and used to quantify relative A20 expression by densitometry, as reported beneath the WB. Densitometry of the bands of interest and was determined as the mean intensity of the areas delineated by Image J, then corrected by the main intensity of the corresponding housekeeping gene band. Fold induction was determined using the non-treated 5 mM D-glucose condition sample as one (1). Data are representative of 3 independent experiments. The cuts between samples reflect the fact that these samples, while on the same gel and same experiment, were not contiguous. (B) Analysis of A20 mRNA levels by real-time PCR. SMC were cultured in 5 or 30 mM D-Glu or mannitol (25 mM Mannitol +5 mM D-GLu), as an osmotic control, and treated with TNF for 1 or 3 h. 18S ribosomal RNA was used to normalize the data. Natural log transformed data (ln) are presented as mean±SEM of 3 independent experiments performed in duplicate. No significant differences (P>0.05) were noted between all groups and at all time-points.

**Figure 2:**
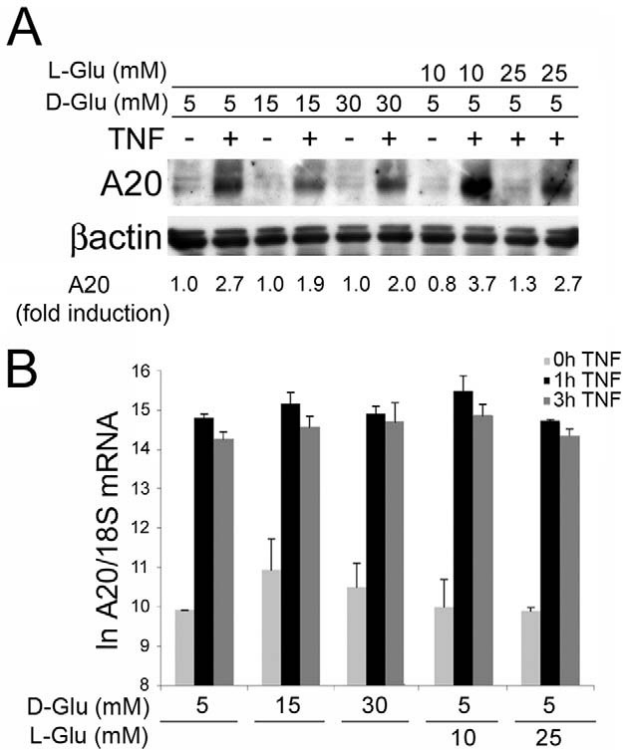
Increasing glucose (D-Glu) concentrations decreases TNF-mediated A20 protein up-regulation without affecting its transcriptional activation in EC. (A) Analysis of A20 expression by WB. EC cultured in medium containing 5, 15 or 30 mM D-glucose (D-Glu) or L-glucose (L-Glu), as an osmotic control, were stimulated with TNF for 6 h̃βactin was checked as loading control and to quantify relative A20 expression by densitometry, as reported below the WB. Densitometry of the bands of interest and was determined as the mean intensity of the areas delineated by Image J, then corrected by the main intensity of the corresponding house keeping gene band. Fold induction was determined using the non-treated 5 mM D-glucose condition sample as one (1). A20 protein migrates as a doublet in EC and hence both bands were scanned. Data are representative of 3 independent experiments. (B) Analysis of A20 mRNA levels by real-time PCR. EC cultured in medium containing 5, 15 or 30 mM D-Glu or L-Glu as an osmotic control, were stimulated with TNF for 1 and 3 h. Expression of 18S ribosomal RNA was used to normalize the expression of A20 mRNA. Natural log transformed data (ln) are presented as mean±SEM of 3 independent experiments performed in duplicate. No significant differences (P>0.05) were observed between all groups and at all time-points.

To evaluate the impact of high glucose on expression of the atheroprotective protein A20, we cultured SMC in physiologic (5 mM) and high (30 mM) D-Glucose (D-Glu), or the respective osmotic controls [25 mM of L-Glucose (L-Glu) or mannitol in addition to the 5 mM D-Glu present in the medium] for 24 h, challenged them with TNF at 200 U/ml, recovered cell extracts 6 h later, and checked for A20 expression by Western blot analysis (WB). In preliminary experiments, we had determined that A20 protein levels peaked 6 h following TNF stimulation in both EC and SMC. In SMC cultured in 5 mM D-Glu or the osmotic controls (L-Glu or Mannitol), we consistently noted 1.5 to 2.0-fold increase in A20 protein levels 6 h following TNF treatment. TNF-mediated upregulation of A20 decreased, by at least 30%–35%, in SMC cultured in 30 mM D-Glu (Figure 1A). In contrast to A20 protein, A20 mRNA significantly increased within 1 h following TNF addition and remained increased at 3 h regardless of the culture medium used, indicating that high glucose did not impact A20 transcription or mRNA stability (Figure 1B). In preliminary experiments, we had determined that A20 mRNA levels peaked 1–3 h following TNF stimulation in EC and SMC. Similar results were obtained in EC. We consistently noted a 2.5 to 3.7-fold increase in A20 protein levels 6 h following TNF treatment in EC cultured in 5 mM D-Glu and the osmotic controls. TNF-mediated upregulation of A20 also decreased by 30% in EC cultured in high glucose (15 and 30 mM D-Glu; Figure 2A), while TNF-mediated upregulation of A20 mRNA was not affected (Figure 2B).

### Hyperglycemia blunts the physiologic increase of A20 protein in the mouse vasculature following an inflammatory insult and in failed vein grafts of diabetic patients

**Figure 3:**
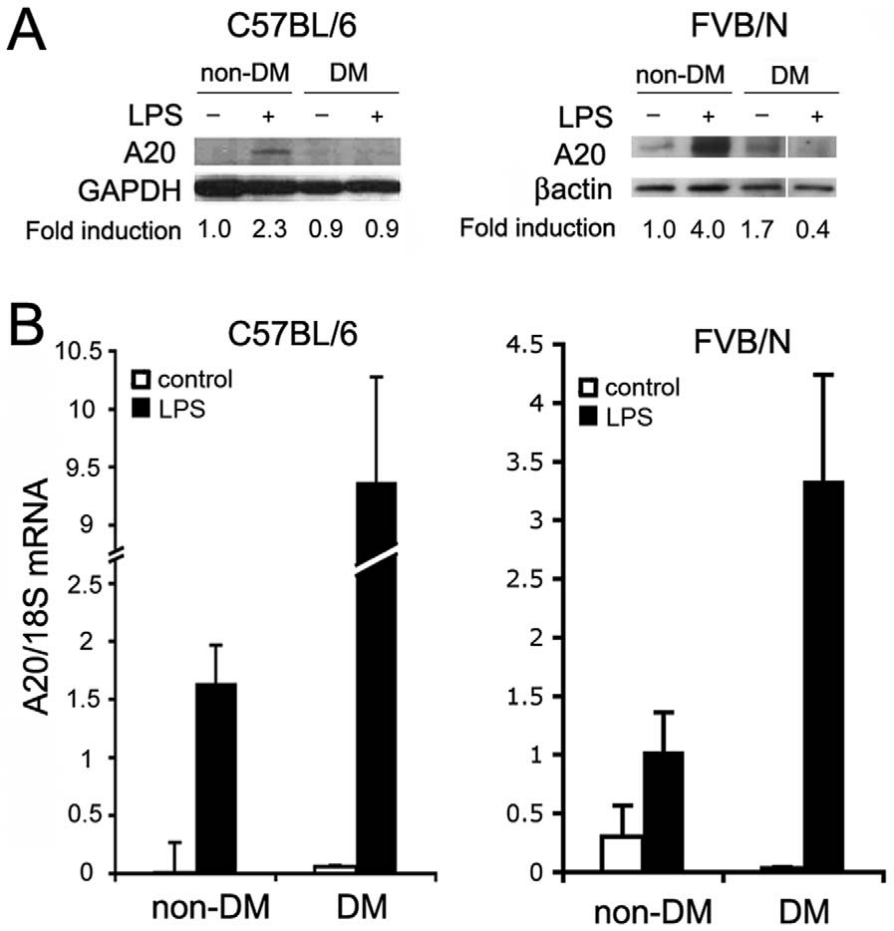
LPS-mediated upregulation of A20 protein expression is blunted in aortae of diabetic, as compared to non-diabetic mice. (A) WB of A20 in abdominal aortae of diabetic and non-diabetic atherosclerosis-prone C57BL/6 and atherosclerosis-resistant FVB/N mice, 8 h after LPS treatment. GAPDH and βactin were used to correct for loading and quantify relative A20 expression by densitometry, as reported below the WB. Data shown are representative of 3 (non-diabetic) and 4 (diabetic) mice per time-point and illustrate the loss of LPS-induced A20 protein in diabetic mice, regardless of strain. The cuts between samples reflect the fact that these samples, while on the same gel and same experiment, were not contiguous. (B) A20 mRNA levels analyzed by real-time PCR 3 to 8 h after LPS injection in mouse abdominal aortae (n = 5 non-diabetic and 7 diabetic mice in C57BL/6 and 3 non-diabetic and 4 diabetic mice in FVB/N). Data shown demonstrates that LPS increases A20 mRNA levels in aortae of diabetic and non-diabetic C57BL/6 and FVB/N, albeit at a greater levels in diabetic mice. Expression of 18S ribosomal RNA was used to normalize expression of A20 mRNA, and the results were presented as mean±SEM of mRNA. Each sample was measured in duplicate.

To check whether hyperglycemia attenuates vascular upregulation of A20 in response to an inflammatory insult, and validate our *in vitro* results, we challenged diabetic and non-diabetic C57BL/6 and FVB/N mice with LPS. These mice strains were chosen because of their described resistance and susceptibility, respectively, to atherosclerosis, based on the function and expression levels of their A20 gene. Indeed, a single point mutation in the C57BL/6 A20 gene decreases its ability to inhibit NF-κB activation, and hence to temper inflammation, rendering these mice susceptible to atherosclerosis. FVB/N mice express a non-mutated form of A20 that exerts a greater NF-κB inhibitory and anti-inflammatory effect, protecting them from atherosclerosis [16], [17]. LPS injection increased A20 protein levels by 2- to 3-fold in aortae of non-diabetic C57BL/6 and FVB/N mice. In contrast, we did not detect any A20 protein in the aortae of LPS-treated diabetic mice despite higher A20 mRNA levels in aortae of diabetic, LPS-treated mice, whether C57BL/6 or FVB/N, compared to non-diabetic mice (Figure 3A & B). These data imply that hyperglycemia decreases A20 expression through translational or post-translational mechanisms independently from transcriptional regulation and genetic polymorphism. As a result both the “functional” i.e. atheroprotective A20 protein of FVB/N mice and the “defective” A20 protein of C57BL/6 mice were equally lost [16].

**Figure 4:**
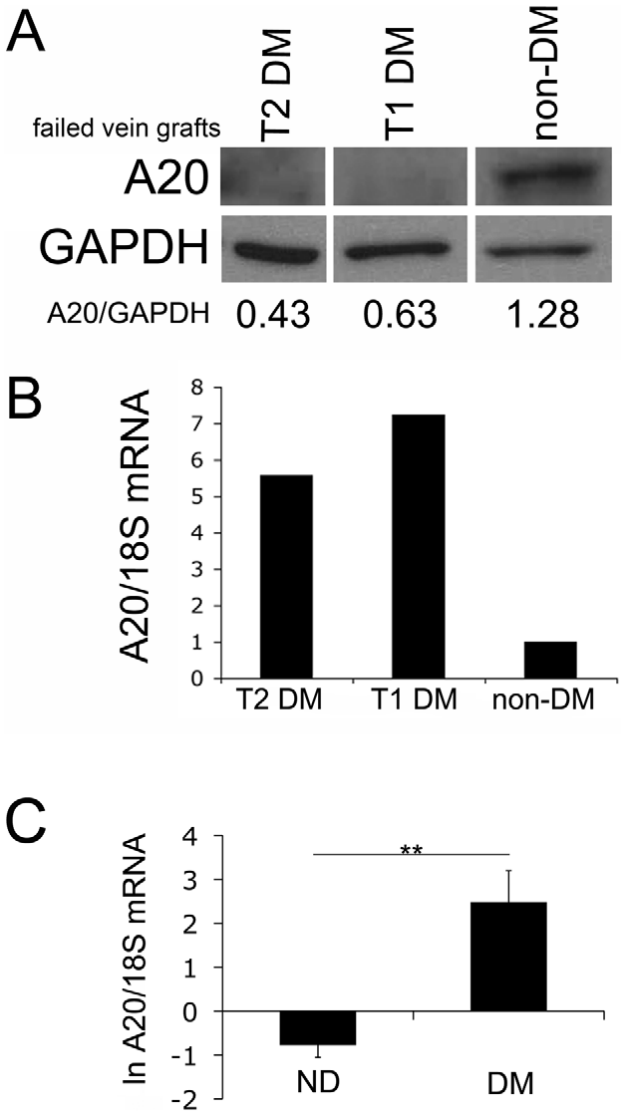
Lower A20 protein levels contrast with increased mRNA levels in failed vein grafts of diabetic patients. (A) A20 protein expression and (B) mRNA levels (real-time PCR, lower panel) in failed vein grafts recovered from non-diabetic (ND) and patients with type I and type II DM (t1 and t2 DM) show lower A20 protein levels in failed vein grafts of diabetic patients as compared to non-diabetic patients, despite significantly higher A20 mRNA levels in these samples. Expression of GAPDH was checked as a loading control and to quantify relative A20 expression by densitometry. A20/GAPDH ratios are listed below the WB. Data shown are representative of 4 patients in each group. Samples were all run on the same gel but are shown as separate because they were not loaded contiguously. (B) A20 mRNA levels of the same failed vein grafts shown in 4A demonstrate increased A20 mRNA levels in failed vein grafts from diabetic vs. non-diabetic patients. 18S ribosomal RNA was used to normalize the data. Data is expressed as fold increase of A20 mRNA using the A20 mRNA level detected in the failed vein graft of the non-diabetic patient as a baseline value. (C) A20 mRNA levels analyzed by real time PCR from failed vein grafts of diabetic (DM) and non-diabetic (ND) patients show significantly higher A20 mRNA in failed vein grafts of diabetic versus non-diabetic patients; **p = 0.0016. 18S ribosomal RNA was used to normalize the data. Natural log transformed data (ln) are presented as mean±SEM of 6 failed vein grafts per group. The 6 failed vein grafts from diabetic patients included 2 type I and 4 type II DM patients. Data is expressed as fold increase of A20 mRNA using the A20 mRNA level detected in the failed vein graft of the non-diabetic patient shown in Figure 1A, as a baseline value.

These data are supported by our clinical observation that almost no A20 protein was detected in failed vein grafts of diabetic patients despite significantly higher A20 mRNA levels in these veins (5.5 to 7.2-fold more). This finding reflects the heightened inflammation that is known to be associated with diabetes, as compared to A20 mRNA levels detected in failed vein grafts from patients suffering from pro-inflammatory and pro-atherogenic conditions other than diabetes (Figure 4A&B). In contrast, A20 protein levels were higher in failed vein grafts from non-diabetic patients (as expected in response to ongoing inflammation within these failed vein grafts) (Figure 4A, n = 4 patients per group), despite lower A20 mRNA levels in these veins, as compared to A20 mRNA levels in the failed veins recovered from diabetic patients (Figure 4B, samples paired with WB & Figure 4C, n = 6 patients per group). Although the pathophysiology of failed vein grafts is different from that of atherosclerosis, this data allowed us to clinically confirm the discrepancy between high A20 mRNA levels and low A20 protein levels in the vasculature of diabetic versus non-diabetic patients.

### A20 is O-GlcNAcylated and ubiquitinated in SMC cultured in high glucose

**Figure 5:**
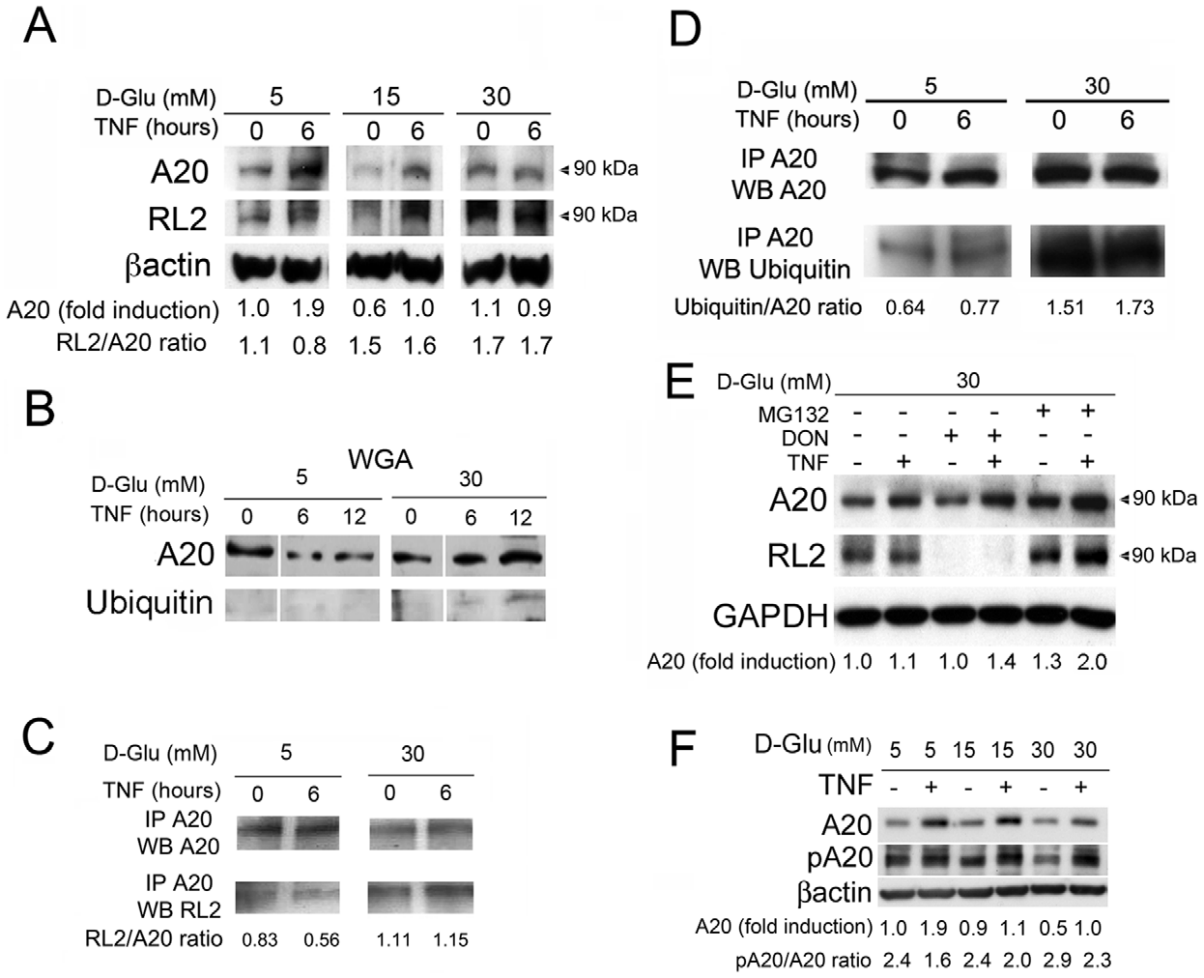
O-GlcNAcylation and ubiquitination of A20 modulate its expression. (A) WB analysis of total A20 and co-immunoblotted, overlapping GlcNAc-A20 (RL-2) in SMC cultured in 5, 15 and 30 mM of D-glucose (D-Glu), and treated or not with TNF for 6 h. β-actin was used as a control for loading. (B) WB analysis of WGA captured proteins from SMC cultured in 5 and 30 mM D-Glu demonstrate the presence of glycosylated (GlcNAcA20), and co-immunoblotted, overlapping, ubiquitinated A20 (Ub-A20). (C) WB analysis of cell lysates immunoprecipitated with the A20 antibody from SMC cultured in 5 and 30 mM of D-Glu and treated or not with TNF for 6 h, and analyzed WB for total A20 and GlcNAc-A20 using the RL2 antibody demonstrate increased GlcNAc-A20 in high glucose medium. (D) WB analysis of cell lysates immunoprecipitated with the A20 antibody from SMC cultured in 5 and 30 mM D-Glu and treated or not with TNF for 6 h, and analyzed by WB for total and Ub-A20 demonstrate increased Ub-A20 in high glucose medium. (E) WB analysis of total and overlapping GlcNAc-A20 (RL-2) in SMC cultured in 30 mM D-Glu and treated with DON (prior to TNF) or MG132 (after TNF). (F) WB analysis of total and phospho-A20 in SMC cultured in 5, 15 and 30 mM D-Glu and treated with TNF for 6 h demonstrated that relative phosphorylation levels of A20 (pA20) were not decreased by high glucose, despite decreased TNF-mediated upregulation of A20 protein in cells cultured in high glucose. GAPDH or βactin was checked as a loading control to quantify A20 expression by densitometry. Corrected A20 fold-inductions are listed below the WB. RL2/A20 and Ubiquitin/A20 ratios were also calculated by densitometry. Data shown in A, C, D, and E are representative of 3 independent experiments. Data shown in B and F are representative of 2 independent experiments.

We searched for glucose-induced PTM that could account for decreased A20 protein levels in cells exposed to high glucose and TNF, focusing on SMC cultures since they are the principal cell type comprising atherosclerotic lesions. Most of these experiments were reproduced at least once in EC. A20 does not contain any N-glycosylation consensus motif [26]. However, we identified several serine and threonine residues that could potentially be O-GlcNAcylated [27]. Immunoblots using the anti-GlcNAc antibody, RL-2, demonstrated a 90-kDa band that overlapped with the band recognized by the anti-A20 antibody in lysates from SMC cultured in 5, 15 and 30 mM D-Glu and treated with TNF for 6 h. This suggested that A20 undergoes modification by O-linked N-Acetylglucosamine under conditions of high glucose (Figure 5A). While TNF-mediated upregulation of total A20 protein decreased in SMC cultured in 15 and 30 mM D-Glu, O-GlcNAcylation of the 90 kDa A20 band, detected in a co-immunoblot using an RL-2 antibody, increased in parallel with increasing glucose concentrations, as determined by RL-2/A20 densitometry ratios. To confirm that A20 was targeted for post-translation O-GlcNAcylation, and given the difficulty of performing immunoprecipitation with the currently available anti-GlcNAc antibodies, namely RL-2, we first mixed cell lysates from TNF-treated SMC cultured in 5 or 30 mM D-Glu with wheat germ agglutinin (WGA) affinity beads. The WGA lectin preferentially binds terminal N-acetyl glucosamine (GlcNAc) structures i.e. both N-linked Asparagine and O-linked serine/threonine residues, but also has high affinity for sialic acid [28]. WB analysis of WGA captured proteins using rabbit anti-A20 polyclonal antibody showed that A20 was one of the WGA-captured proteins (Figure 5B). Since WGA columns are relatively non-specific, the same cell lysates were also immunoprecipitated (IP) with the rabbit anti-A20 antibody (IP-A20). WB of IP-A20 samples with anti-A20 and RL-2 antibodies indicated that A20 was O-GlcNAcylated in both 5 and 30 mM D-Glu, albeit at a greater ratio in cells cultured in HG. The ratio of GlcNAc-A20 over total A20 (determined by densitometry) increased by 1.4 to 2 fold in SMC cultured in 30 mM D-Glu (Figure 5C).

To determine whether increased O-GlcNAc of A20 contributed to its decreased protein levels, we probed for accelerated degradation of GlcNAcA20 by the ubiquitin/proteasome pathway. Using WB with an anti-ubiquitin antibody, we identified a faint positive band migrating at 90 kDa (likely Ub-A20) that was detected in WGA-captured proteins from TNF-treated SMC, cultured in 30 mM D-Glu (Figure 5B). To further explore this observation, we immunoprecipated cell lysates from TNF-treated SMC cultured in 5 and 30 mM D-Glu with the rabbit anti-A20 polyclonal antibody. WB of IP-A20 samples, using anti-ubiquitin and anti-A20 antibodies demonstrated that high glucose increased the ratio of ubiquitinated over total A20 (UB-A20/total-A20) by 2 to 2.5-fold before and 6 h after treatment with TNF (Figure 5D). Notably, data shown in Figure 5C & 5D demonstrate remarkable similarity between GlcNAc-A20/total-A20 and Ub-A20/total-A20 ratios, suggesting that these PTM could be interdependent.

**Figure 6:**
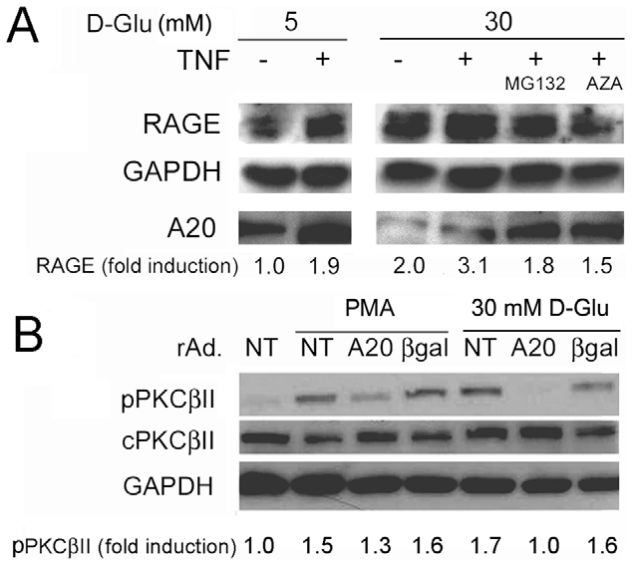
Restoring A20 levels reverts glucose-mediated upregulation of RAGE and phosphorylation of PKCβII. (A) WB analysis for RAGE and A20 expression in SMC cultured in 5 or 30 mM D-Glu for 24 h and treated with TNF in the presence or absence of 20 mM of Azaserine (prior to TNF) or 10 mM of MG132 (following TNF). Corrected RAGE fold-inductions are listed below the WB. The RAGE protein is detected as a doublet as a result of pre and post-N-glycosylated form of the protein. Both bands were used for densitometry evaluation. (B) WB analysis of phospho-PKCβII (pPKCβII) and total (c) PKCβII in NT SMC, and in SMC transduced with rAd.A20 or rAd.βgal, and treated with PMA or challenged with 30 mM D-Glu for 1 h. Data shown in A and B are representative of 3 independent experiments. NT = non-transduced cells. GAPDH was used as loading control to quantify the relative expression of RAGE and pPKCβII by densitometry.

To that effect, inhibiting glutamine fructose-6 phosphate amidotransferase (GFAT), which catalyzes the first and rate-limiting step in the formation of glucosamine and hexosamine products[29], by pre-treating SMC cultured in 30 mM D-Glu with its specific inhibitor 6-diazo-5-oxo-norleucine (DON, 50 µM) 1 h prior to TNF treatment, or addition of the proteasome inhibitor MG132 (10 µM) 1 h following TNF treatment, restored A20 protein expression (Figure 5E). WB using RL-2 confirmed that pre-treatment with DON inhibited A20 O-GlcNAcylation, while treatment with MG132 allowed for the accumulation of an O-GlcNAcylated A20. Similar results were obtained when using another GFAT inhibitor, Azaserine (20 µM) (Figure 6A).

Since A20 function is, at least in part, regulated by phosphorylation of Ser381 [30], we investigated whether high glucose affects TNF-induced phosphorylation of A20 in SMC by WB analysis using specific anti-phospho A20 antibody. High glucose had no effect on TNF-mediated A20 phosphorylation, as it did not decrease the ratio of phosphorylated to total A20 levels (Figure 5F). Similar results were obtained in EC (data not shown).

### Inhibition of proteasome activity or overexpression of A20 inhibits high glucose-mediated upregulation of RAGE and phosphorylation of PKCβII

AGE/RAGE signaling is one of the key pathways implicated in the pathophysiology of DV [31], [32]. We confirmed by WB that RAGE levels increased in SMC cultured in 30 mM D-Glu and that this increase was further amplified by TNF (Figure 6A). Restoring A20 protein by inhibiting O-GlcNAcylation (with Azaserine), or blocking the activity of the proteasome (with MG132) significantly inhibited upregulation of RAGE by high glucose and TNF (Figure 6A). Similar results were obtained when A20 protein levels were restored by transducing SMC with rAd.A20 (Figure 6B). This indicates that considerable overexpression of A20 overwhelms the glycosylation/ubiquitination machinery, maintaining adequate levels of functional A20, as gauged by its ability to decrease RAGE upregulation.

Activation of PKCβII in response to high glucose is another pathway involved in promoting DV [6]. By WB, we determined that PKCβII was phosphorylated, and thus activated in non-transduced (NT) and rAd.βgal-transduced SMC challenged for 1 h with 30 mM D-Glu, similarly to what we observed upon treatment with 1 µM phorbol 12-myristate 13-acetate (PMA). High glucose and PMA-mediated phosphorylation of PKCβII was inhibited or even abolished in A20-overexpressing SMC (Figure 6C).

### Expression of A20 in aortic arches protects diabetic ApoE-null mice from accelerated atherosclerosis

**Figure 7:**
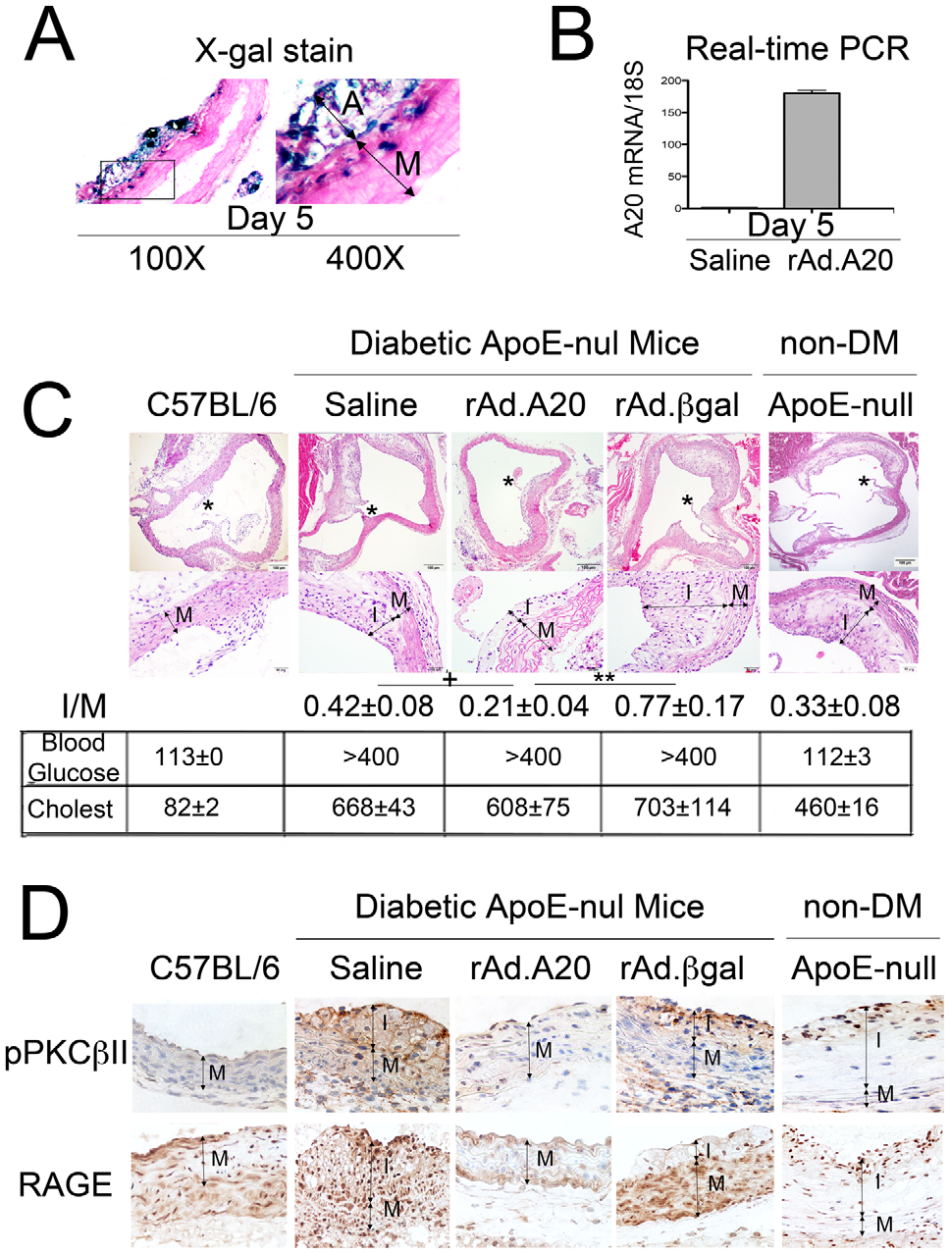
Expression of A20 in the ascending aorta and aortic arch of diabetic ApoE-null mice prevents the development of atherosclerotic lesions by inhibiting PKCβII phosphorylation and blunting the induction of RAGE. (A) Transgene expression was confirmed by X-gal staining in rAd.βgal-transduced vessels 5 days following transgene delivery (n = 3 mice/group) and demonstrate the expression of the transgene in medial SMC (M) as well as the adventitia at the level of the aortic root, albeit not in all cells. Image amplification 100× and 400×. (B) A20 expression was verified by real time RT-PCR in two rAd.A20-transduced vessels, using human A20 specific primers that do not recognize mouse A20. Our data indicate significant expression of human A20 in aortic roots of rAd.A20 but not saline treated mice. Results are shown as average± SE. (C) H&E stained aortic root sections at the level of the first coronary from 20 week-old diabetic ApoE-null mice treated with saline, rAd.A20 or rAd.βgal. Images are shown at 100× and 400× as indicated by the scale bar. The asterisk indicates the level of the first coronary branch, Arrows define the intima (I) and the media (M). ApoE-competent, non-diabetic C57BL/6 and non-diabetic ApoE-null mice were used as controls. Blood glucose and cholesterol levels (cholest) are listed below the sections. *P<0.05 compared to saline, ** P<0.01 compared to rAd. βGal. Data shown are representative of 4 to 6 mice per group. I/M ratios were calculated after analysis of 10 serial sections per vessel. (D) phospho-(p)PKCβII (5 days) and RAGE (14 days) immunostaining in aortic arches 5 and 14 days after transgene delivery. Data shown in C are representative of all sections analyzed (n = 3 mice per group, 2–3 sections analyzed per vessel). Image amplification 200×.

**Figure 8:**
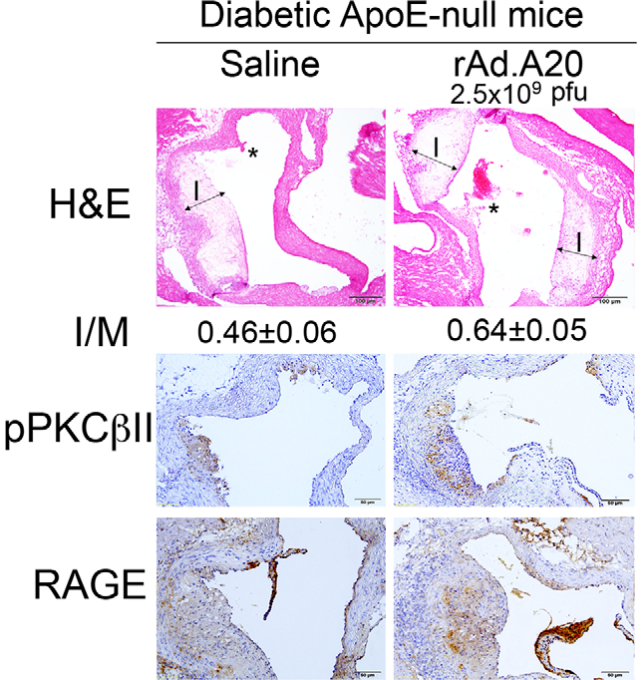
Intravenous administration of rAd.A20 to diabetic ApoE-null mice fails to protect them from accelerated atherosclerosis. Evaluation of I/M ratio on H&E stained aortic arch sections from 20-week old mice (8 weeks following intravenous administration of 2.5×10^9^ pfu/mice of rAd.A20 or saline) demonstrated comparable intimal lesions in saline and rAd.A20-treated mice (n = 6 mice/group). Images are shown at the level of the first coronary artery at 100× magnification. The asterisk indicates the level of the first coronary artery branch. Arrows define the intima (I). Immunohistochemistry analysis of RAGE and phospho-PKCβII (pPKCβII) demonstrate equally intense staining in the neointima of saline and rAd.A20-treated mice. Image amplification 400×.

As stated earlier, diabetes accelerates and aggravates atherosclerosis in ApoE-null mice [33]. Having demonstrated that diabetic mice express lower A20 levels under inflammatory conditions, we questioned whether restoring A20 levels in the vasculature could prevent accelerated atherosclerosis in diabetic ApoE-null mice. We overexpressed A20 in aortic arches of diabetic ApoE-null mice six weeks after documentation of diabetes (early stages of atherosclerotic lesion development) by administering rAd.A20 (5×10^9^ pfu) through the left ventricle. This route of adenovirus delivery leads to preferential expression of the transgene in medial SMC and the adventitia, peaking at 5 days following administration, as shown by X-gal staining in mice treated with rAd.βgal (Figure 7A). We confirmed A20 overexpression in aortic arches of mice treated with rAd.A20 by real-time PCR (Figure 7B). We sacrificed mice at 20 weeks and recovered their aortic arches for evaluation. Ten serial sections 50 µm apart were evaluated per vessel. There were prominent atherosclerotic lesions in saline and rAd.βgal-treated vessels showing significant expansion of the intima, foam cell infiltration and reduction of the media. I/M ratios reached 0.42±0.08 and 0.77±0.17, respectively (Figure 7C). A20 expression significantly inhibited the development of atherosclerotic lesions with calculated I/M ratios reaching 0.21±0.04 (P = 0.024 vs. saline and P = 0.006 vs. rAd.βgal) (Figure 7C). The protective effect of A20 against DV did not correlate with an effect of A20 on blood glucose or cholesterol levels, which remained comparable among groups (Figure 7B). As expected, hyperlipidemia was worsened by diabetes (P<0.01; Figure 7C). Two control groups included non-diabetic ApoE-null mice that showed fewer cellular lesions as well as C57BL/6 mice that displayed normal vascular architecture. Importantly, the protective effect of A20 against DV required local vascular expression of the transgene. Intravenous administration of rAd.A20 to diabetic ApoE-null failed to protect from accelerated atherosclerosis, ruling out a systemic effect of A20 on the observed outcome (Figure 8).

### A20 protects against accelerated atherosclerosis of DM by inhibiting PKCβII phosphorylation and attenuating RAGE upregulation *in vivo*

By immunohistochemistry (IHC), we detected numerous EC and intimal SMC as well as a few medial SMC that intensely stained for phospho-PKCβII in mouse aortic atherosclerotic lesions 5 days following saline or rAd.βgal treatment. Overexpression of A20 in aortic arches decreased the intensity of phospho-PKCβII immunostaining and limited its expression to only a few EC (Figure 7D). Similar results were obtained 14 days after gene transfer (data not shown). We did not detect phospho-PKCβII positive cells in aortic arches of ApoE-competent C57BL/6 mice and detected only faint immunostaining in organized atherosclerotic lesions of non-diabetic ApoE-null mice.

There was significant RAGE expression in aortic atherosclerotic lesions of diabetic Apo-E-null mice (EC and medial SMC) even prior to transgene delivery (data not shown). RAGE immunostaining intensified and extended to developing neointimal SMC and foam cells 14 days following saline and rAd.βgal administration (Figure 7D). In contrast, RAGE immunostaining was blunted in A20-expressing aortic arches and was similar to staining detected in non-diabetic C57BL/6 mice. We also identified RAGE positive EC and neointimal SMC in atherosclerotic lesions of non-diabetic ApoE-null mice, but the number of positive cells and intensity of staining were lower in comparison to diabetic ApoE-null mice. Intravenous administration of rAd.A20 did not affect the intensity or distribution of phospho-PKCβII and RAGE immunostaining in the atherosclerotic lesions of diabetic ApoE-null mice, further indicating that the atheroprotective effect of A20 requires local vascular expression (Figure 8).

## Discussion

Several studies have identified poor glycemic control and heightened TNF levels as potential risk factors for vascular complications in DM [34], [35]. It seems well established that standard glycemic control (hemoglobin A1C between 7 and 7.9%) is beneficial for reducing micro and macrovascular complications of diabetes [36]. However, the ACCORD study has clearly shown that a very strict glycemic control (hemoglobin A1C levels lower than 6%), while still beneficial for reducing the risk of diabetic retinopathy, increases the incidence of fatal cardiovascular events for reasons that are still poorly understood [37], [38]. Accordingly, we set our goals to define the molecular targets of high glucose in EC and SMC that support increased atherosclerosis, and determine ways that could rescue his phenotype, independently from glycemic control. We demonstrated that hyperglycemia amplifies the risk for atherosclerosis through decreased vascular expression of the physiologic “atheroprotective” protein A20. A20 upregulation in response to inflammatory signals was greatly reduced in EC and SMC cultured in high glucose, and in vessels of hyperglycemic mice and patients. Decreased A20 levels in response to high glucose did not result from transcriptional repression. A20 mRNA levels were even greater in vessels of hyperglycemic vs. non-hyperglycemic mice and patients, which is consistent with the fact that NF-κB activation is amplified by high glucose, increasing transcription of downstream NF-B-dependent A20 [39]. Rather, high glucose decreased A20 expression at the post-translational level by promoting its O-GlcNAcylation and ubiquitination. We detected GlcNAc-A20 in cells cultured in physiologic glucose concentrations, but its levels were amplified by high glucose concentrations, likely as the result of increased flux through the hexosamine biosynthetic pathway [40]. We also demonstrated, *in vitro*, that O-GlcNAcylation and ubiquitination of A20 increased in parallel, which suggested that these PTM could be, related either directly or indirectly. Accordingly, A20 was shunted away from proteasomal degradation when O-GlcNAcylation was inhibited, allowing recovery of A20 protein levels in the face of high glucose and inflammation (mimicked by TNF). Moreover, we restored sufficient A20 protein levels by inhibiting the activity of the 26S proteasome, which led to the accumulation of a functional GlcNAc/Ub A20, as demonstrated by decreased expression of the NF-κB dependent pro-inflammatory gene, RAGE, a direct target of the NF-κB inhibitory protein A20 [41]. Our result demonstrating that O-GlcNAcylation promotes the ubiquitination of certain proteins agrees with work by Guinez et al. It shows that conditions that promote protein O-GlcNAcylation also enhance protein ubiquitination; conversely, inhibiting O-GlcNAcylation reduces protein ubiquitination in HepG2 cells [42]. Our data focusing on A20 in primary SMC echoes their conclusion that O-GlcNAcylation regulates ubiquitination in a sequential and dependent manner. However, with A20 being a target for both PTM, our current data does not allow us to distinguish whether O-GlcNAcylation of A20 is required or is merely a marker for its subsequent ubiquitination. In the former scenario, one may envision that O-GlcNAcylation of A20 could modify the availability and/or function of its own ubiquitin ligase domain to promote its autoubiquitination [22]. In the latter scenario, A20 O-GlcNAcylation may be a simple indicator for O-GlcNAcylation levels, targeting other ubiquitin editing enzymes (i.e. ubiquitin activating enzyme E1) to increase their activity and hence ubiquitination of their target proteins including, A20 [42]. Future work in the laboratory is aimed at clarifying this issue by generating GlcNAc-resistant (once the identity of O-GlcNAcylated residues in A20 are identified) and/or ubiquitin ligase deletion A20 mutants.

Our data contrast, however, with other results suggesting that promoting O-GlcNAcylation protects some proteins such as Sp1 from proteasomal degradation[43]. Sp1 protein stabilization in conditions that favor protein O-GlcNAcylation likely stems from Sp1's own O-GlcNAcylation, which hinders its ubiquitin-independent, proteasomal degradation[44], [45], [46]. Further studies are required to determine whether opposite effects of O-GlcNAcylation on Sp1 and A20 in terms of promoting/inhibiting proteasomal degradation may relate to the ubiquitin-dependent (A20) versus independent (Sp1) proteasomal degradation of these proteins.

A more generalized defect in proteasome function as a result of O-GlcNAcylation of the Rpt2 protein on the 19S cap proteasome has also been proposed as a potential mechanism causing decreased proteasomal degradation of Sp1 in high glucose conditions [44], [45]. Although we did not directly test the impact of O-GlcNAcylation on proteasome function in EC and SMC, we demonstrated increased proteasomal degradation of A20 in these cells when cultured in high glucose. This data does not support the presence of a major defect in proteasome function in our system, unlike those of NRK and breast cancer cell line MDA 468 [45]. While we recognize that we cannot rule out proteasome dysfunction in EC and SMC cultured in high glucose, we excluded a level of dysfunction that would significantly decrease the degradation of ubiquitinated proteins, such as A20. Importantly, our results were validated in animal vessels and in patients' vein graft samples.

Protein phosphorylation is another PTM that interacts with O-GlcNAcylation, competing for the same residues to regulate protein function [47]. Our data failed to demonstrate any effect of high glucose upon phosphorylation of Ser381 a residue required for A20's NF-κB inhibitory function [30].

It is well established that hyperglycemia results in O-GlcNAc mediated PTM that alters the expression/function of atheroprotective proteins, accelerating the development and progression of DV [48]. O-GlcNAcylation of the atheroprotective protein eNOS interferes with its phosphorylation by AKT, blunting its activity and decreasing NO bioavailability [13], [14]. Also, increased O-GlcNAcylation of Sp1 decreases its degradation thereby increasing transcription of the pro-atherogenic gene MCP-1 [40]. We present novel evidence that the anti-inflammatory and atheroprotective A20 protein is another target for O-GlcNAcylation and have yet to demonstrate whether this PTM is a marker or a prerequisite for the increased degradation of A20 in high glucose/hyperglycemia.

Our data are particularly interesting in light of a tag polymorphism identified in the human A20 locus, in which minor alleles were associated with lower A20 mRNA and 3-fold increased incidence of coronary artery disease in well controlled type II diabetics[49]. Patients with poor glycemic control showed similar incidence of coronary artery disease regardless of A20 polymorphism. This apparent discrepancy is easily clarified by our results demonstrating that high glucose/hyperglycemia induces the degradation of the A20 protein, nullifying its atheroprotective effects despite adequate mRNA transcription.

Under conditions that allow the accumulation of A20 in the vasculature, this protein protects from DV by inhibiting the damaging signals triggered by high glucose, namely RAGE upregulation and PKCβII phosphorylation. In SMC challenged by high glucose, we showed a remarkable reciprocal correlation between RAGE and A20 expression. This is consistent with RAGE being a NF-κB-dependent gene whose expression is induced by high glucose-mediated activation of NF-κB [50]. Accordingly, RAGE upregulation was amplified when the levels of the NF-κB inhibitory protein A20 were decreased in cells exposed to high glucose [51], while RAGE induction by high glucose/cytokines was inhibited upon restoring A20 levels, either by rAd-mediated overexpression or by inhibiting proteasome activity. A20 also inhibited the phosphorylation of PKCβII, which is preferentially activated in the vasculature of diabetic animals [52]. Decreased A20 levels in vessels of diabetic patients may account for the amplified and prolonged PKCβII activation demonstrated in DM. We are investigating the molecular basis for inhibition of PKCβII phosphorylation by A20.

Blockade of RAGE by soluble RAGE or treatment with PKCβantagonists reduces macro and microvascular complications of DM [33], [53]. Therefore, it is not surprising that overexpression of A20, which attenuated both RAGE expression and PKCβII activation in aortic arches of diabetic ApoE-null mice, protected from accelerated atherosclerosis despite persistent hyperglycemia and aggravated dyslipidemia. While phospho-PKCβII is pathognomonic for glucose-mediated vascular damage, increased RAGE immunoreactivity was also detected in atherosclerotic lesions of non-diabetic ApoE-null mice, consistent with increased AGE/RAGE levels in response to the oxidative stress of dyslipidemia [32], [54]. This may account for the dramatic atheroprotection provided by A20 in the diabetic ApoE-null mouse model via down-regulation of hyperglycemia-dependent and hyperglycemia-independent effectors of atherosclerosis.

**Figure 9:**
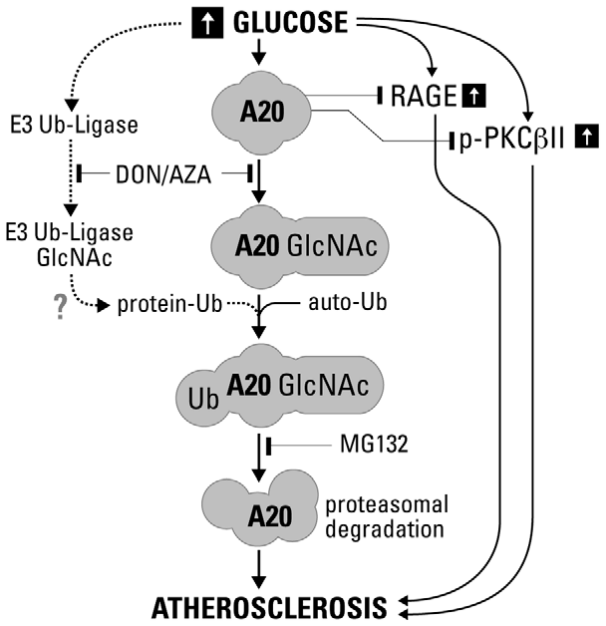
High glucose promotes A20 O-glycosylation, ubiquitination and proteasomal degradation in EC and SMC. High glucose increases protein O-GlcNAcylation, including that of A20 and possibly other E3 Ubiquitin ligases. This leads to increased A20 ubiquitination either through auto-ubiquitination or increased activity of other O-GlcNAcylated E3 ubiquitin ligases. This targets A20 for degradation in the proteasome. Blockade of O-GlcNAcylation using DON, upstream of A20 Ubiquitination, or inhibition of proteasome activity, using MG132, downstream of A20 ubiquitination would inhibit its proteasomal degradation, restoring its expected protein levels.

From a basic science standpoint, these data propose a novel mechanism by which O-GlcNAcylation promotes ubiquitination and proteasomal degradation of A20 (Figure 9). Clinically, our results identify the loss of the anti-inflammatory and atheroprotective protein A20 in DM as a key pathogenic contributor to DV, given the protective effects of this protein against the deleterious signals of high glucose in the vasculature [55], [56] (Figure 9). From a therapeutic standpoint, our data offer convincing proof that A20 or possibly GlcNAc-resistant A20-based gene therapy delivered to the vessel wall may have clinical applicability in post-angioplasty restenosis, and for modification of vein grafts for coronary artery and peripheral bypass surgery.

## Methods

### Ethics Statement

The ethical committee of the Beth Israel Deaconess Medical Center approved all experimental projects. All animal use is in compliance with all current US government regulations concerning the care and use of laboratory animals. There are no veterinary concerns related to the use of mice as performed in this paper. Supervision of animal care was conducted by staff members of a fully AAALAC accredited facility headed by Dr. Garibaldi. All personnel handling the animals followed a specialized training prior to starting. The BIDMC has been certified for Animal Welfare Assurance. The number is A3153-01, expiring on 2/28/2014. The approved protocol number is #091-2006.

As for the discarded vein grafts from patients, the recovery of this material follows all recent NIH/ADAHMA policy guidelines concerning the inclusion of women and minorities in human research. Recovery of vessels from patients in the operating room was approved by the Institutional Review Board of the BIDMC. This study has been approved by CCI with waiver of Consent and Authorization. These tissues, which are otherwise discarded, were strictly used for research purpose. The IRB approval number for this protocol is 2001-P-002020/7; BIDMC Legacy #: W-91-0012-EX. The BIDMC holds a Federal-Wide Assurance for human subjects: FWA00003245.

### Reagents

Human coronary artery EC and SMC were obtained from Lonza (Portsmouth, NH) [18], [20]. Human recombinant TNF was purchased from R&D Systems (Minneapolis, MN). The proteasome inhibitor MG132, O-diazoacetyl-L-serine (Azaserine), a glutamine analog that inhibits GFAT, and the GFAT inhibitor 6-diazo-5-oxo-norleucine (DON) were from Sigma (St Louis, MO).

### Recombinant adenoviral vectors

We generated a recombinant adenovirus encoding A20 (rAd.A20) from an expression plasmid (gift from Dr. V. Dixit, Genentech Inc., CA). Control rAd. β-galactosidase (rAd.β gal) was a gift from Dr. R. Gerard (University of Texas SW). SMC and EC were transduced at a multiplicity of infection (MOI) of 500 and 100, as described [18], [20].

### Immunoprecipitation and Western blot

Immunoprecipitation (IP) was performed with homemade rabbit anti-A20 polyserum or with agarose bound wheat germ agglutinin (WGA, Vector laboratories, Burlingame CA) on spin-X columns. Western blot (WB) analysis utilized rabbit anti-A20 polyserum as well as antibodies against the following: human A20, RAGE and N-Acetylglucosamine (RL-2) (AbCam, Cambridge, MA), phospho-A20 (gift of Dr. L. Cantley, BIDMC), phospho-PKCβII Serine-660 (BioSource International, Camarillo, CA), total (c-) PKCβII and βactin (Santa Cruz, Biotechnology, Santa Cruz, CA), ubiquitin C-terminal Hydrolase, and GAPDH (Chemicon International Inc. Temecular, CA). After scanning of all films, densitometry of the bands of interest and of the corresponding house keeping gene was performed using the ImageJ software. In brief, the image was first inverted then an exact markdown of the white bands was performed using the hand-drawing tool The mean intensity of the delineated area was measured, then corrected by the main intensity of the corresponding house keeping gene band. Fold induction was determined using the basal condition sample as one (1).

### Real-time RT-PCR

RNA was extracted using RNeasy Mini Kits (Qiagen, Valencia, CA) and cDNA synthesized using Superscript III First-Strand Synthesis System (Invitrogen, Carlsbad, CA). Probes and primers for human and mouse A20 were commercially purchased (Assays on demand, Applied Biosystems Assays-on-demand Inc., Foster City, CA). Expression of A20 mRNA was normalized to expression of 18S rRNA.

### Determination of glycosylation

SMC were harvested by sonication in lysis buffer comprising 5 mM HEPES pH 7.4, 10 mM NaCl, 0.1 mM DTT, 0.5 mM EDTA, 5% glycerol supplemented with a protease inhibitor cocktail (Roche Diagnostics Corporation, USA). Glycosylation was determined by immunoprecipitation with agarose bound wheat germ agglutinin (WGA, Vector laboratories, Burlingame CA) on spin-X columns followed by WB analysis using rabbit anti-A20 polyclonal antibody[57].

### RNA and protein extraction from mice aortae and patients vein grafts

C57BL/6 and FVB/N mice were rendered diabetic by treatment with five daily IP injections of streptozotocin (STZ, 60 mg/kg, Sigma) in citrate buffer (0.05 M; pH 4.5). Control mice received citrate buffer. STZ-treated mice with blood glucose >350 mg/dl at two consecutive readings were considered diabetic. LPS (50 µg/mouse, Sigma) was administered IP and the aortae were harvested 3 and 8 h later for RNA (RLT lysis buffer) and protein (RIPA buffer supplemented with protease inhibitors) extraction. Failed vein grafts from patients were recovered from discarded tissue at the time of surgery, as approved by the BIDMC Institutional Review Board (IRB), with waiver of Consent and Authorization. These tissues, which are otherwise discarded, were processed for RNA and protein extraction.

### Accelerated atherosclerosis in diabetic ApoE-null mice

Male homozygous ApoE-null mice on the C57BL/6 background (Jackson Laboratories, Bar Harbor, ME) were maintained on a regular chow diet. At six weeks of age, mice were rendered diabetic with STZ. At 12 weeks of age, 5×10^9^ plaque forming units (pfu) of rAd or saline were injected into the left ventricle of anesthetized animals. Mice treated with similar dose and schedule, but using the intravenous route, were included in the study to rule out a systemic effect of the transgene on disease incidence. Eight weeks after administration of the rAd, hearts and aortae were removed “en bloc” following perfusion fixation in 10% formalin. Serum cholesterol levels were measured at Cornell University Diagnostic Laboratory (Ithaca, NY). Aortic arches were also retrieved at day 0, 5, and 14 after adenoviral delivery and snap frozen in Tissue-Tek. The BIDMC Institutional Animal Care and Use Committee approved all procedures.

### Histology and immunohistochemistry

As stated earlier the heart and the aortic arch were removed en bloc… Paraffin embedded tissue sections were sectioned serially in 50 µM increments starting from the apex of the heart, and stained with hematoxylin/eosin (H&E). Intima/media (I/M) ratios were measured in a blinded fashion by 2 investigators (GVS and RP) on 10 consecutive serial section/sample, using ImageJ 1.62 software. Lesions were demonstrated throughout the ascending aorta starting at the beginning of the aortic root i.e the aortic annulus and extending to the sinotubular junction. All images shown were taken at the level of the first coronary artery. Frozen sections were fixed with 2% paraformaldehyde (for RAGE) or acetone (for phospho-PKCβII) prior to immunostaining and processing as described [18]. β-galactosidase expression was detected by X-gal staining. Immunohistochemistry (IHC) sections were analyzed by EC, OK and CF.

### Statistical Analysis

Quantitative data were expressed as mean±standard error of mean (SEM). Statistical analysis was performed using analysis of variance (ANOVA) followed by Tukey-Kramer multiple comparisons test or unpaired two-tailed student-t-test using GraphPad InStat software. Some data were Natural Log transformed prior to analysis. P<0.05 was considered to be statistically significant.
